# Minimum electric‐field gradient coil design: Theoretical limits and practical guidelines

**DOI:** 10.1002/mrm.28681

**Published:** 2021-02-09

**Authors:** Peter B. Roemer, Brian K. Rutt

**Affiliations:** ^1^ Roemer Consulting Lutz Florida USA; ^2^ Department of Radiology Stanford University Stanford California USA

**Keywords:** asymmetric gradient, E‐field, electric field, folded gradient, gradient coil, head gradient, peripheral nerve stimulation, PNS

## Abstract

**Purpose:**

To develop new concepts for minimum electric‐field (E‐field) gradient design, and to define the extents to which E‐field can be reduced in gradient design while maintaining a desired imaging performance.

**Methods:**

Efficient calculation of induced electric field in simplified patient models was integrated into gradient design software, allowing constraints to be placed on the peak E‐field. Gradient coils confined to various build envelopes were designed with minimum E‐fields subject to standard magnetic field constraints. We examined the characteristics of E‐field‐constrained gradients designed for imaging the head and body and the importance of asymmetry and concomitant fields in achieving these solutions.

**Results:**

For transverse gradients, symmetric solutions create high levels of E‐fields in the shoulder region, while fully asymmetric solutions create high E‐fields on the top of the head. Partially asymmetric solutions result in the lowest E‐fields, balanced between shoulders and head and resulting in factors of 1.8 to 2.8 reduction in E‐field for x‐gradient and y‐gradient coils, respectively, when compared with the symmetric designs of identical gradient distortion.

**Conclusions:**

We introduce a generalized method for minimum E‐field gradient design and define the theoretical limits of magnetic energy and peak E‐field for gradient coils of arbitrary cylindrical geometry.

## 1 INTRODUCTION

Gradient coil and amplifier technologies have developed rapidly over the past few decades, such that MRI gradient performance is now more limited by peripheral nerve stimulation (PNS) than by hardware capabilities. The rapid switching of strong gradients, required for high‐speed and high‐resolution imaging, gives rise to electric fields (E‐fields) in the human body that can depolarize nerves, with peripheral nerves typically being much more sensitive than central nerves. Low‐level PNS is considered safe, but uncomfortable levels must be avoided. Because of PNS, present‐day high‐performance body gradients often operate below their hardware limits for sequences that require high amplitudes and/or switching rates.^1^, ^2^ In theory, as these amplitudes or switching rates increase, there is a risk of cardiac stimulation, although cardiac thresholds are typically much higher than PNS thresholds.^3^ Widely adopted regulatory guidelines such as those found in International Electrotechnical Commission 60601‐2‐33^4^ require that scanners operate at or below population‐average PNS thresholds and well below cardiac stimulation thresholds.

The primary determinant of the PNS threshold for a given gradient coil is its linear region length along the z‐axis.^2^ Whole‐body gradient coils with longer linear regions expose larger areas of the body to rapidly switching magnetic fields, inducing higher E‐fields. Several special‐purpose shorter‐body gradient concepts were developed to more efficiently generate gradient fields, while at the same time lifting PNS restrictions.^5^, ^6^ Toward this same end, dedicated head gradient coils were developed.^1^, ^7^, ^8^, ^9^, ^10^, ^11^, ^12^, ^13^ Several head gradient designs and experimental constructions have been demonstrated in recent times, and experimental PNS studies have confirmed their significantly improved PNS performance compared with body gradient coils.^14^, ^15^, ^16^

Despite these recent advances in gradient coil technology, including important efforts to design and build specialty gradient coils with improved PNS characteristics, there is no generalized strategy for PNS‐optimal gradient design. Such a strategy requires accurate prediction of PNS thresholds for a population of human subjects before coil construction, as well as the ability to control and iteratively optimize PNS thresholds at the gradient design stage. The International Electrotechnical Commission standard specifies that the PNS threshold for safe operation be calculated using the peak E‐field on the surface of a uniform‐interior body model.^4^ Motivated by this, we recently developed highly efficient computational methods to evaluate the spatial distribution of E‐fields over realistically sized uniform‐interior body models. Using these methods, we have shown that population‐mean PNS thresholds can be predicted accurately (to within 20% mean absolute error) from peak surface E‐fields, for a range of gradient coils including two very different head gradient coils.^17^

Prior work has proposed the incorporation of E‐field calculations or PNS estimation into the gradient design process.^18^, ^19^, ^20^, ^21^, ^22^, ^23^, ^24^, ^25^ Expanding on these efforts, we integrate our computationally efficient E‐field methods into a gradient design algorithm that is then used to evaluate both theoretical and practical limits of minimum E‐field gradient design. We believe our approach is an improvement for finding globally optimal solutions over a wide range of anatomical dimensions and gradient topologies, and for providing a solid conceptual understanding of the theoretical limits of minimum E‐field gradient design. Our approach is also compatible with established MRI safety standards,^4^ in which E‐field limits and simplified body models are required unless otherwise justified.

## 2 METHODS

### 2.1 Electric field calculation

Gradient design is a relatively mature field of MR engineering. However, present‐day gradient design algorithms do not typically incorporate constraints on the E‐fields that produce PNS in human subjects, and this has limited our ability to find minimum‐E‐field gradient designs. However, as described here, it is relatively straightforward to add an E‐field constraint to the gradient design, given the availability of analysis software that can compute E‐fields for a given body model and arbitrary gradient magnetic field. In principle, any method that computes the E‐field (such as existing finite‐element software) can be used, but a high‐speed method will allow for more comprehensive analyses. We integrated our efficient methods for calculating the E‐field within and on the surface of realistically sized uniform body models^17^ into an existing gradient design algorithm. We then used this new capability to survey the minimum E‐field versus stored energy relationship for the entire human MRI gradient design domain (both body and head size), spanning conventional designs that do not enforce any E‐field constraints to heavily E‐field‐constrained designs. In so doing, we reveal the theoretical as well as practical limits of minimum‐E‐field gradient design for a given patient geometry.

### 2.2 Body models

For the current work, we use the same family of body models used in our E‐field and PNS analysis work.^17^ These body models use ellipsoidal and elliptic cylindrical shapes for head, neck, shoulders, and torso sections; because of their simplicity, they are ideal for computationally efficient evaluation of E‐fields. The key physical dimensions of these body models match anatomical dimensions of adults extracted from the Humanscale anthropometric reference material.^26^ A total of six body models are defined and used for our calculations; these six models represent the 2.5th, 50th, and 97.5th percentile male and female adult populations. Table 1 lists the dimensions for these six body models.

**TABLE 1:** Dimensions in mm of body models used in the calculation of the E‐fields, covering 95% of the adult male and female populations, and derived from the Humanscale manual^26^

Item	Male			Female		
	2.5%	50%	97.5%	2.5%	50%	97.5%
x ellipse radii, shoulder	203.0	225.0	246.5	183.0	203.0	225.0
x ellipse radii, neck	53.5	58.5	63.5	51.7	55.9	60.2
x ellipse radii, head	72.5	77.5	82.5	72.5	72.5	77.5
y ellipse radii, torso	98.0	114.5	136.0	97.0	107.0	117.0
y ellipse radii, neck	57.2	61.2	66.7	51.7	55.9	60.2
y ellipse radii, head	92.5	98.0	104.0	91.5	92.5	98.0
z lengths (ellipse radii), body endcap	50.0	50.0	50.0	50.0	50.0	50.0
z lengths, body straight section	488.0	457.0	424.0	455.0	424.0	396.0
z lengths, body/neck transition	150.5	164.0	173.0	142.5	157.0	170.5
z lengths, neck/head transition	97.3	101.0	108.0	90.3	94.0	97.8
z lengths (ellipse radii), top of head	97.3	101.0	108.0	90.3	94.0	97.8
z brain center (relative to top of head)	−97.3	−101.0	−108.0	−90.3	−94.0	−97.8
z eye center (relative to top of head)	−102.0	−112.0	−119.0	−102.0	−112.0	−119.0

### 2.3 Gradient design with added E‐field constraints

As is typical in gradient coil design, a set of arbitrary basis functions, each of fixed shape and unknown amplitude, is used to describe the gradient coil winding pattern. The design problem is cast as finding these basis function amplitudes, such that either stored energy or power dissipation are minimized subject to a set of linear constraints. Constraints are placed on positional error (linearity error), pixel size error (uniformity error), and gradient strength at a set of points spanning the desired imaging volume. Additional constraints can be placed on other quantities such as force, torque, eddy currents, current density, concomitant fields, and, as we describe here, E‐field.

Because our goal in relation to PNS optimization is to constrain the peak E‐field on the surface of a body model positioned in the gradient coil, we compute the E‐field distribution for each gradient coil basis function at a set of points on the surface of the body model using the methods described in Roemer et al.^17^ Given the linearity of Maxwell’s equations, the E‐field can be expressed as a linear combination of magnetic‐field basis functions. There is flexibility in the choice of the basis set within certain requirements. Each basis function should have fixed geometry with unknown amplitude; this makes the problem linear. A linear combination of basis functions should be capable of representing an arbitrary distribution. Considering these requirements, we chose sines and cosines as our basis set for several reasons. A 2D Fourier series painted over the gradient‐coil surface, where the unknowns are the coefficients, is a general set of basis functions. This basis set is a good match to the angular symmetry of most gradient coils and can efficiently represent the z‐distribution of typical gradient coil windings. Additional details concerning the choice of gradient‐coil basis functions can be found in the Supporting Information.

Defining X as the vector of unknown basis function amplitudes, the solution can be cast into a quadratic programming problem that minimizes energy:

(1) $$X^{T}MX=\text{min}$$

subject to the inequality and equality constraints

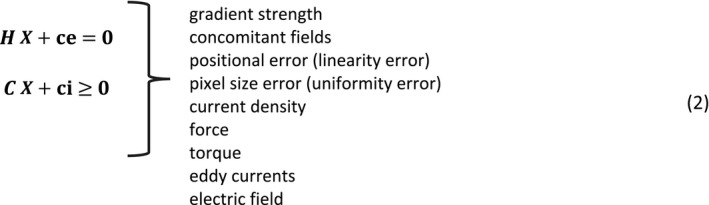

where **M** denotes a mutual inductance matrix, and M_i,j_ represents the mutual inductance between basis functions *i* and *j* of unit amplitude, and is readily computed from the known current distribution of each basis function. Alternatively, **M** can be replaced by a mutual resistance matrix to minimize power dissipation, or a weighted combination can be used. We use a mutual inductance matrix for the present work.

The matrices **H** and **C** contain the linear constraint equations: one column for each unknown basis function, and one row for each constraint. The desired gradient strength is achieved by enforcing an equality constraint equation at the isocenter, where the value of **ce** is the negative of the desired gradient strength, and the column entries in the **H** matrix are the gradient strengths for each basis function of unit amplitude. A B_0_ concomitant field constraint can be added that requires the corresponding transverse field component to be zero at the isocenter. Other field constraints such as positional error and pixel size are handled in a similar manner. Within the imaging volume, inequality constraints are placed at multiple points over the imaging volume on the pixel size error and the fractional positional error defined as

(3) $$\text{Pixel}\;\text{Size}\;\text{Error}\overset{\text{def}}{=}\frac{1}{G}\frac{\partial B_{z}}{\partial n}‐1$$

(4) $$\text{Fractional}\;\text{Positional}\;\text{Error}\overset{\text{def}}{=}\frac{1}{R}\left(\frac{B_{z}}{G}‐n\right)$$

where *n* is the desired gradient direction (X, Y, or Z); *G* is the desired gradient strength at isocenter; *R* is the radius of the imaging volume; and *B_z_* is the longitudinal component of field. For the analysis here, we use values of 50% for pixel size (uniformity) error and 16.5% for fractional positional (linearity) error over a 260‐mm‐diameter spherical volume. These typical values (which correspond to design parameters used for a recently described head gradient^14^) allow the pixel size to vary by a maximum of a factor of 2 and the maximum positional error to be 21.5 mm at the periphery of the imaging volume.

Force and torque constraints can be added using the known main B_0_ field distribution, integrating over the current distribution for each basis function and entering the results into matrix **H** or **C**. One row is added for each direction of force or torque, to obtain an overall magnitude constraint. Eddy currents are computed in a similar manner; the step response for time‐dependent eddy currents on the surface of a conducting cylinder—typically the thermal shield in the magnet—is computed at multiple points in space and time. Each point in space and time is entered as a separate inequality constraint equation. It is usually sufficient to constrain eddy currents at time = 0 following a step change in gradient current, reducing the size of the problem.

Finally, and of primary significance to the present work, E‐field constraints can be handled in a similar manner to those for force and torque. At gradient frequencies of interest, the time evolution of E‐fields in human tissues is short, and therefore proportional to instantaneous slew rate and not the detailed waveform shape in time. Thus, given a body model, the E‐field is computed from each gradient coil basis function for unit slew rate at a series of points over the body surface, such as using the methods we have described recently.^17^ Calculation at points on the interior of the model is not required, as the maximum magnitude always occurs on the surface of a uniform body model (see Supporting Information). A magnitude constraint is created on the surface by placing a series of rotated inequality constraints on the vector E‐field tangent to the surface. For example, we choose 32 directions at each point spaced over 360º, which constrains the magnitude to within 0.5% of the desired value.

### 2.4 Gradient winding regions

Three regions are defined that serve as spatial boundaries to confine the gradient windings within which we determine the current distribution that minimizes magnetic energy subject to all field constraints. This generalized approach allows globally optimal solutions to be determined, limited only by a specified volumetric boundary.

**FIGURE 1:**
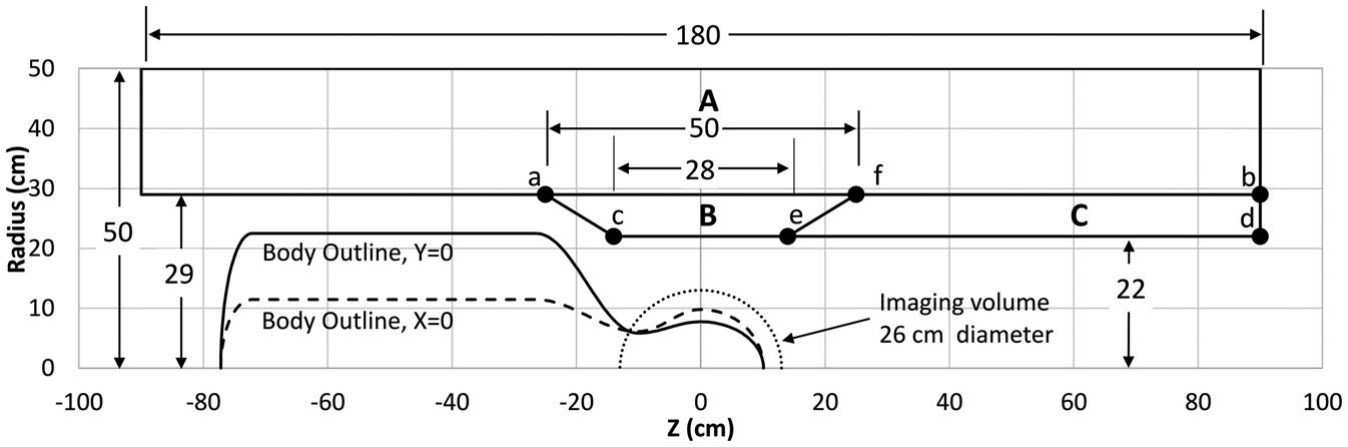
Outline of the body in relationship to the imaging volume and regions where gradient currents are permitted to flow, designated by the capital letters A, B, and C. The lowercase letters of a, b, c, d, e, and f designate points connecting line segments that define surfaces where currents can flow and are used for some example solutions. The body outline shown is for 50th percentile male

Figure 1 shows the three regions, indicated as A, B and C, within which gradient windings are permitted, along with their relationship to the imaging volume and the body model outline. Gradient windings are allowed within single or combination regions, such as within A only, B only, combination of B and C, or combination of A, B and C. Region A by itself encompasses all whole‐body gradient coils. Region B by itself has the smallest size and might be used for symmetric short head gradient coils, folded or conventional, but could also be used for compact asymmetric designs. The combined BC region defines the space typically occupied by existing asymmetric head gradient coils. Region ABC spans the greatest spatial extent, encompassing both whole‐body and head gradient coils, and therefore the most general and unconstrained. For this reason, ABC region results will have the lowest bounds on magnetic energy and E‐fields, and we use this region to establish the theoretical lower limits that all gradient coils that fit within this build envelope must satisfy. Within all of these regions the azimuthal variations of gradient current density are limited to sine, cosine or axisymmetric, depending on the gradient axis, but the current density is otherwise unconstrained. Small letters a‐f in Figure 1 define surfaces on which currents are limited for some solution families, for comparison with volumetric solutions.

The curl and divergence of **B** are both zero external to the build envelope; therefore, each Cartesian component of **B** satisfies Laplace’s equation outside the build region. The equivalence principle in electromagnetics means that magnetic fields external to a region produced by an arbitrary distribution of currents internal to that region can be represented equivalently and uniquely by currents on its surface.^27^ As a result, in practice, our optimization proceeds by allowing arbitrary currents to flow only on the surfaces of described volumetric regions, further simplifying the analysis without loss of generality.

Our methods produce minimum magnetic energy solutions for a given constraint on maximum E‐field (E_max_) on the body model surface. This procedure is repeated over a range of E_max_ constraints, starting with no constraint, and progressively reducing the constraint amplitude. The resulting family of curves represents the fundamental relationship between minimum achievable E_max_ and magnetic energy.

## 3 RESULTS

**FIGURE 2:**
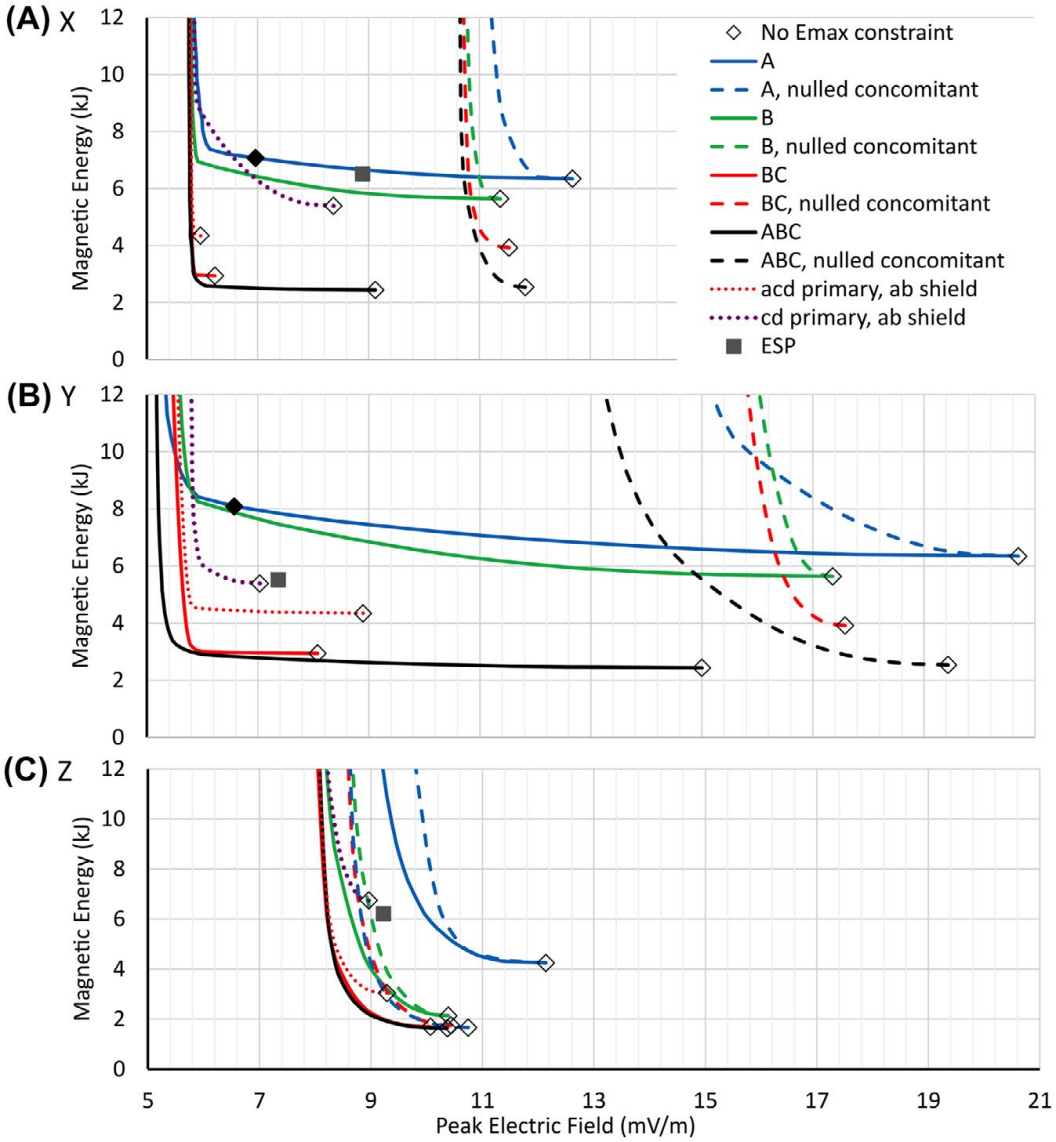
Solution results for X (A), Y (B), and Z (C) gradient directions, all using 50% male body model. The electric field (E‐field) constraint is progressively lowered while the energy is minimized. Magnetic Energy (y‐axis) is per unit gradient strength of 1 T/m, and Peak Electric Field (x‐axis) is per unit slew rate of 1 T/m/s. The black, red, green, and blue solid and dashed lines represent currents flowing in regions ABC, BC, C, and A, respectively. The dashed lines are solutions in which no B_0_ concomitant field offset was allowed at gradient isocenter. The red dotted lines correspond to a primary winding placed on surface acd and a shield winding on surface ab. The purple dotted lines correspond to a primary winding on surface cd and shield winding on surface ab. The open diamonds are with no E‐field constraint. The solid filled diamonds denote E_max_‐constrained solutions near the knee of the A region curves

Figure 2 shows the general form of the E_max_ versus magnetic energy curves for all configurations studied, and for each of the X, Y, and Z coils. These L‐shaped curves demonstrate that the maximum E‐field on the body can, in some cases, be reduced substantially without dramatic increase in magnetic energy. However, below a certain constrained E‐field value, characterized by the knee of the curve, the magnetic energy begins to rise rapidly. There is a well‐defined minimum achievable E‐field shown by the vertical asymptote of each curve.

Figure 2 shows the results for four different volumetric regions (A, B, BC, and ABC) and two surface regions (acd and cd) using the 50th percentile male body model. For the A, B, BC, and ABC curves, no other constraints such as force, eddy current, or current density limitations are applied; therefore, the resulting curves represent minimum‐E_max_ versus minimum‐energy boundaries that no practical solution can exceed (ie, no designs are possible to the left of or below these curves). Specifically, the solid black curves in Figure 2 represent region ABC, yielding both the lowest E_max_ and the lowest energy solutions of all regions. All gradient coils with windings that fall within or on the surface of volume ABC must result in E_max_ and magnetic energy to the right of and above this solid black curve. As this boundary is restricted to smaller subregions (eg, restricted to A [blue curves], BC [red curves], or B [green curves]) regions), the horizontal asymptote, representing the lower limit of magnetic energy, increases.

We see immediately that all X coils are bounded by a single vertical asymptote, representing a single fixed lower limit of E‐field; this observation is also approximately true for Y and Z coils. This tells us that with appropriate E‐field constraints incorporated into the gradient design process, minimum‐E_max_ coils designed over a wide range of sizes and shapes converge to a relatively fixed optimal PNS behavior, a remarkable conclusion.

A second set of dashed curves in Figure 2 shows the impact of setting all vector components of the gradient field to zero at isocenter, thereby removing any B_0_ concomitant field for the X and Y gradient coils or zeroing the field offset for the Z coil. The results show a significant increase in minimum E_max_ by forcing concomitant fields to zero: The vertical asymptote is approximately doubled for X gradients and tripled for Y gradients, although the penalty for Z gradients is much less extreme (< 10%). We note that z‐symmetric coils naturally exhibit zero concomitant fields. These results show that z asymmetry in transverse gradient coils is necessary for reducing the E‐field.

Regions A and B are geometrically symmetric about the z = 0 plane, with minimum energy solutions (assuming no E‐field constraint) yielding z‐symmetric field solutions and zero B_0_ concomitant fields. Hence, the open diamond starting point (representing the minimum energy solution with no E‐field constraints) for the solid and dashed curves (in both black and green cases) coincide. The symmetry is broken by the addition of a patient model with an E‐field constraint.

The knee in the curves marks the point where magnetic energy, current density, and winding complexity increase rapidly as E_max_ is driven to lower values. These complexities render unrealizable or impractical any gradient designs on the vertical portions of the curves to the left of the knee. Examination of winding patterns indicates that solutions that exhibit energy increases > 25% (or even less depending on coil type) above the global minimum energy become unrealizable (eg, demonstrating two or more “eyes” per half coil [four or more total eyes for an x or y coil] in the fingerprint wire patterns).

The red and purple dotted curves in Figure 2 represent two practical cases in which the primary coil currents are restricted to a single inner surface near the patient and the shield coil currents to an outer cylinder, while at the same time adding torque and eddy current constraints. Line segments acd of Figure 1 represent a primary winding surface consisting of a cone and a cylinder, whereas line segment ab represents its cylindrical shield winding; this yields designs similar to those proposed by previous literature^8^, ^22^ and results in the red dotted curves in Figure 2. A second case is defined by restricting primary currents to the cylinder represented by line segment cd and shield currents to the cylinder ab; this yields designs similar to those proposed by previous literature^28^ and produces the purple dotted curves in Figure 2. For both configurations, the eddy current surface is a 1‐m‐long, 620‐mm‐diameter cylinder, centered about the gradient isocenter and placed outside the shield winding of the gradient coil. The time = 0 eddy current error is constrained to a peak deviation less than 0.1% of the ideal gradient field on the surface of a 20‐cm‐diameter volume, corresponding to 100 µT for a 1 T/m step change in gradient strength. The torque constraint is set to zero for a uniform z‐directed B_0_ main magnetic field. There is no net force for a perfectly uniform main B_0_ field in all calculations.

As expected, adding eddy current and torque constraints yields solutions to the right of and above the solid red curves of region BC. The eddy current and torque constraints demand additional magnetic energy, but interestingly, as the E‐field constraint is progressively applied, the acd solution (cone/cylinder surface, dotted red curve) converges rapidly to the same asymptotic minimum E_max_ as the BC volume solution (solid red curve). This is expected, because the field on the patient side of the coil determines the E‐field limits, and the cone/cylinder combination spans the same overall z‐extent as region BC with a similar solid angle, as viewed from the imaging volume. The dotted purple curve further limits the primary currents to the inner cylindrical section cd and shows significantly slower convergence toward the asymptotically minimum E_max_, a result of the windings not being permitted onto the conical section defined by ac, which reduces the degrees of freedom for E‐field minimization.

The solid gray squares in Figure 2 show the point solution for the ESP asymmetric gradient coil, whose design closely approximates that of the GE Healthcare (Waukesha, WI) HG2 prototype head gradient^14^; we have recently evaluated the E‐field and PNS characteristics of the ESP gradient coil.^17^ This coil was designed within the BC build envelope and used similar distortion, eddy current, and torque constraints, as described previously, but was further restricted to all‐cylindrical winding surfaces, and no E_max_ constraints were imposed. As expected, the E_max_ and magnetic energy characteristics fall to the right of and above the solid red curve (region BC) in Figure 2, and near the open diamond at the end of the dotted purple curve (cd/ab solution).

### 3.1 Detailed analysis of region A

It is instructive to compare two solutions for region A with and without E‐field minimization, to gain a conceptual understanding of the essential differences between E‐field minimized and nonminimized designs. Due to the symmetry of region A about the z = 0 plane, solutions with no E‐field constraint are naturally symmetric with no B_0_ concomitant field. The addition of an E‐field constraint on an asymmetric body model breaks this symmetry. We find that E‐field reduction requires the addition of a concomitant field component. The open diamond at the right end of the blue curve in Figure 2 marks the lowest‐energy solution for A‐region coils, which is a naturally symmetric solution with no E‐field constraint. The solid diamond on the same curve is close to the knee point, where E_max_ is reduced markedly, but before large increases in magnetic energy and before the winding pattern has developed higher‐order complexity. The solid diamond is at the approximate location where the number of solution eyes in each half coil is ≤ 2 for x and ≤ 3 for y.

**FIGURE 3:**
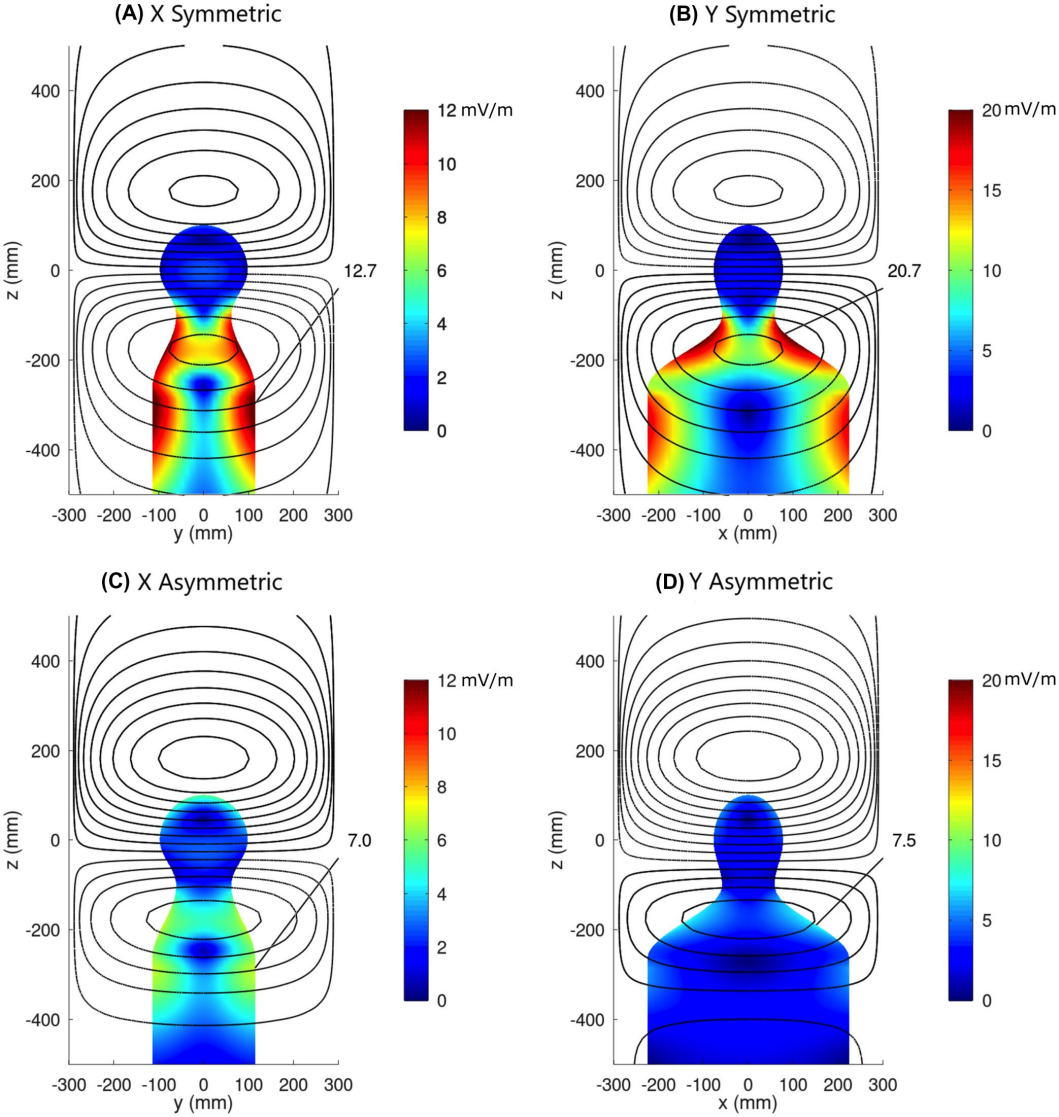
X and Y gradient solutions with and without E_max_ optimization for the 50% male body model. The color scale is the magnitude of the E‐field in units of mV/m for unit slew rate of 1 T/m/sec. A,B, top row: No E_max_ optimization, showing a symmetric gradient solution corresponding to the open diamond on the blue curve of Figure 2A. C,D, bottom row: Optimized solutions corresponding to the solid diamond of Figure 2B, showing a factor of 2.8 decrease in the E‐field. Note that that E‐field color scales are different for the X and Y gradients. The wire plots correspond to the projection of the current distributions onto the surface of region A

Figure 3 shows the surface E‐fields for the X and Y gradient coils of these A‐region designs, with an overlay of the associated winding patterns. The top row shows results with no E‐field constraint, which exhibits a naturally symmetric solution corresponding to the open diamond on the blue curve of Figure 2. The X‐gradient and Y‐gradient windings are identical; however, the Y coil induces larger E‐fields due to the greater cross‐sectional diameter of the body in the left–right direction. The bottom row shows the minimum‐E_max_ designs; the winding patterns for X and Y coils are not identical to each other, but in both cases result in significantly reduced E‐field magnitudes compared with the unconstrained E‐field designs in the top row.

Comparing the top and bottom rows of Figure 3, it is apparent that the strength (wire density) of the winding pattern “eye” below the patient’s head has been reduced, while the strength of the eye above the head has been increased. This leads to a reduction of the time‐varying magnetic flux below the head (which generates circulating currents in the body) and a corresponding increase above the head. The smaller anatomical cross‐section in the head region compared with the shoulder region allows higher fluxes for the same induced E‐field. The optimized solutions demonstrate greatly reduced E‐field magnitudes with equal E‐field magnitudes at the shoulders and head; this “balanced” E‐field solution is a natural outcome of our E_max_‐constrained design strategy.

**FIGURE 4:**
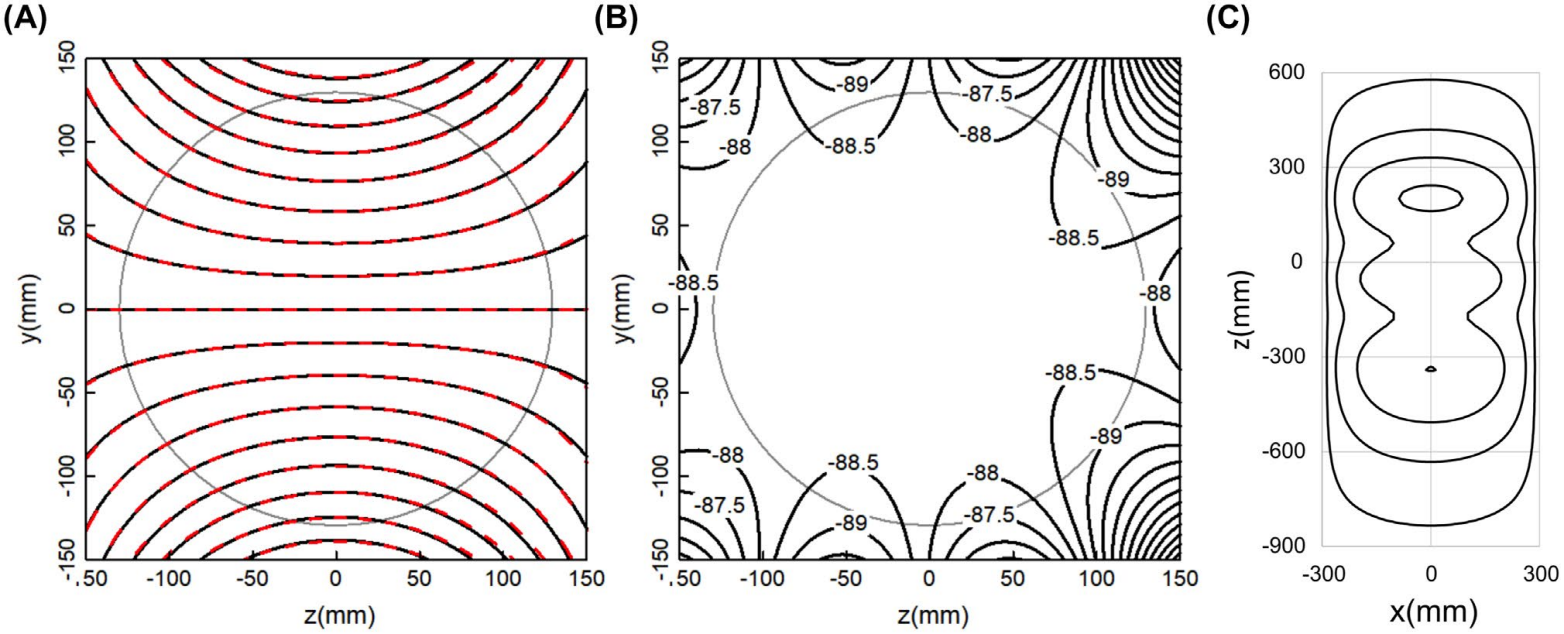
A, A region contours of constant Bz for Y gradient coil, with (red) and without (black) E_max_ optimization, showing that gradient distortion is virtually identical despite the peak E‐field being reduced by a factor of 2.8 for the Y coil. B, Difference in the B_y_ components of the gradient field with versus without E‐field optimization. Values are normalized to the gradient strength yielding units of length (mm) and show a highly uniform concomitant y‐directed field added to the optimized (asymmetric) solution. C, Wire pattern representing the difference between optimized (asymmetric) and nonoptimized (symmetric) solutions with same current scaling as Figure 3. Similar results are obtained for the X gradient coil

Figure 4A compares the z component of the magnetic field for the Y gradient coil of Figure 3, with and without E‐field minimization. It is remarkable that the gradient distortions are virtually identical for the two solutions, yet the minimum‐E_max_ solution has 2.8‐fold reduction in E_max_ with only 30% increase in magnetic energy. Similar observations are made for the X gradient, with 1.8‐fold reduction in E_max_ and only 12% increase in energy.

The key difference between the E‐field minimized and nonminimized solutions is the addition of a uniform concomitant B_0_ field component in the x or y direction. Figure 4B shows a difference plot of the concomitant B_y_ field, normalized to gradient strength (units of millimeters), which is responsible for the reduction in E‐field. Figure 4C shows the winding that would need to be added to the Y symmetric coil (Figure 3B) to obtain the winding pattern of the Y asymmetric coil (Figure 3D), and shows the fundamental difference between the two solutions. The winding in Figure 4C produces a uniform field transverse to the main B_0_ field, resulting in a concomitant component with additional magnetic stored energy. Conceptually, this can be thought of as a second coil, which if activated, converts the symmetric coil into an asymmetric coil with reduced E‐field but otherwise identical distortion. The added concomitant field in Figure 4B is highly uniform with a 0.85% variation over the 26‐cm imaging region, resulting in minimal distortion of the desired gradient field; this concomitant field can be compensated with a phase/frequency offset correction by the scanner. Figure 4B shows that the average concomitant field of 88.5 mm within the imaging region can be directly compared with the z_0x_ defined by Meier et al,^29^ in which a value of 127 mm was reported for an asymmetric head gradient. The z_0x_ for both the ESP and HG2 gradients are approximately 120 mm^30^; these are both fully asymmetric designs (single eye per half coil with all return currents flowing above the head), similar to the original concept defined by Roemer.^12^ The X coil of Figure 3 requires a smaller concomitant field (z_0y_ = 57.8 mm) than the Y coil, reflecting a decreased need to pull E‐field off the torso region due to the smaller extent of the body in the anterior–posterior direction.

### 3.2 Body‐size dependence

**FIGURE 5:**
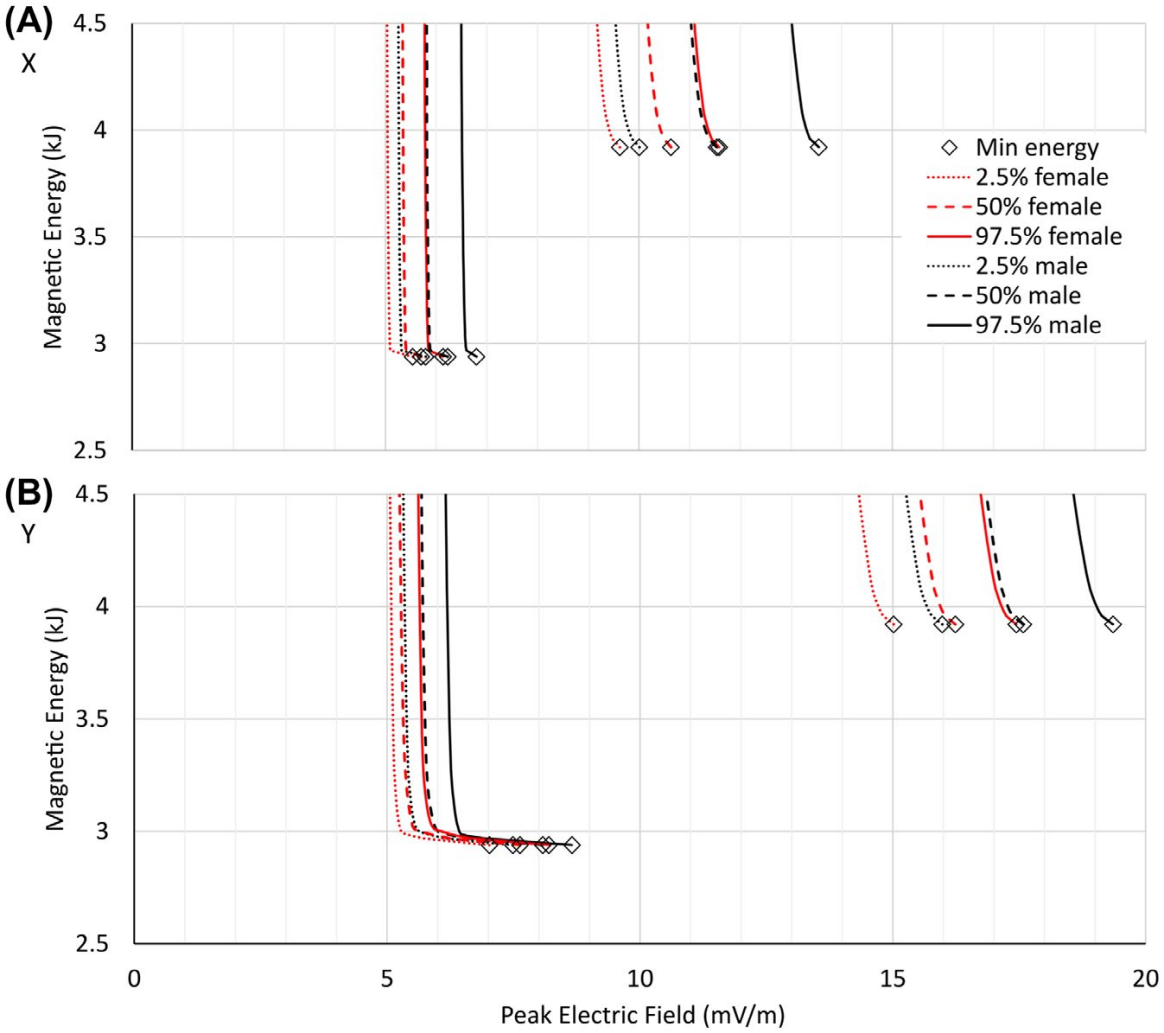
Electric field versus energy‐dependence curves for the six body models from Table 1 for X (A) and Y (B) gradient directions. The group of curves to the right corresponds to solutions in which the B_0_ concomitant field has been nulled, whereas the group of curves to the left corresponds to solutions with nonzero concomitant field

To study the influence of body‐size variation on head gradient performance, we examined region BC solutions, the region normally occupied by head coils. Figure 5 shows the dependence of the E_max_ versus energy across the six body models of Table 1. The right group of curves represents solutions with nulled B_0_ concomitant field, while the left group allows the B_0_ concomitant fields to float to the optimal solution. Not unexpectedly, larger anatomy results in higher values of E_max_ (hence lower PNS thresholds). In addition, the minimum‐E_max_ designs with optimized (nonzero) concomitant field result in lower energy, lower E‐fields, and a smaller variation in the minimum E_max_ across the body‐size range. This can be understood by recognizing that optimized solutions reduce E‐fields in the shoulder region at the expense of increased E‐field on the head. Across the human population, shoulder dimensions are more variable than head dimensions, hence the reduced spread in E_max_ for an optimized design.

Also note that the minimum energy solutions with no E‐field constraint and with no concomitant field constraints, marked by the open diamonds on the left group of curves, are not only lower energy solutions than the concomitant field–constrained solutions, but they are also lower E_max_ solutions. This is understood by recognizing that minimum energy solutions are inherently asymmetric because region BC is asymmetric, resulting in solutions that naturally spread currents above the head, forming a partially asymmetric coil with lower E‐fields.

### 3.3 Body coil E‐field minimization

The addition of concomitant fields to reduce the E‐fields on the shoulders is the fundamental mechanism that allows large reductions in E_max_ for head imaging applications. However, whole‐body gradient coils generally permit patients to be positioned anywhere along the z‐axis. Body gradients designed to minimize E‐fields for head imaging, but that still permit imaging of other anatomical regions, can actually increase E‐fields for other patient positions when compared with conventional symmetric designs. To show this, consider gradients designed with two different imaging region diameters, in both cases with and without E‐field constraints. The first diameter was 26 cm, appropriate for dedicated head imaging. The second imaging region diameter was 40 cm, which is smaller than present‐day whole‐body systems but could represent an appealing design, optimized for head imaging yet with a large enough FOV to provide useful imaging of other portions of the body. For this analysis we reduced the 50% male body section x and y ellipse radii to 200 mm and 100 mm, respectively, to better approximate the population‐averaged torso size (compared with the larger shoulder sizes used for our evaluation of our head gradient designs).

**FIGURE 6:**
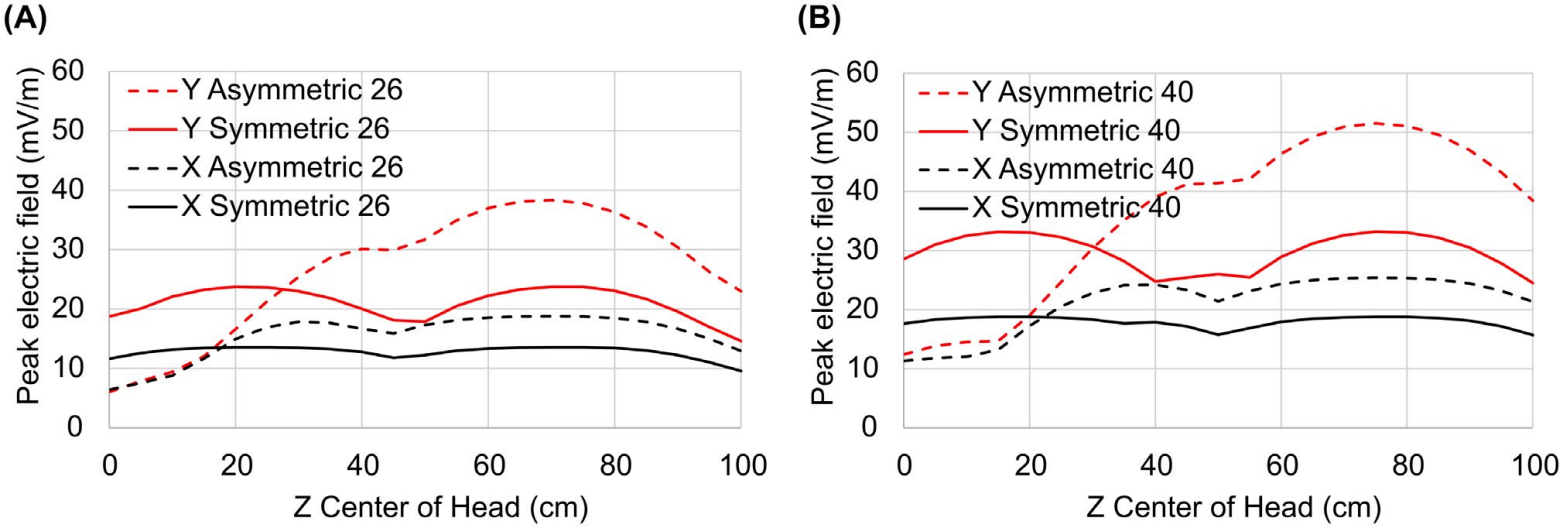
Peak E‐field as a function of body position within the gradient coil. A, The 26‐cm‐diameter imaging region. B, The 40‐cm‐diameter imaging region. Z = 0 corresponds to the center of the head at isocenter. Asymmetric solutions have lower E‐field for head imaging but increased E‐field for body z‐positions > 20‐30 cm into the bore

Figure 6 plots the maximum E‐field found on the body surface as the z‐center of the head is moved away from isocenter and into the gradient coil. The solid curves correspond to conventional minimum‐energy solutions (without E‐field constraints), naturally resulting in z‐symmetric designs. The dashed curves correspond to minimum‐E_max_ designs for a head‐centered body model, resulting in asymmetric designs. With the patient head at isocenter (z = 0), even these large A‐region gradient designs demonstrate minimum‐E_max_ solutions with large (1.5‐fold to 3‐fold) reductions in E_max_. However, as the patient is moved away from head‐centered position and into the magnet (z > 0), E_max_ values for the asymmetric solutions increase faster than for the symmetric solutions, and for z > 30 cm these E_max_ values actually exceed those induced by conventional symmetric gradient designs. This shows that the PNS behavior of the minimum‐E_max_ body gradient solutions, while greatly improved for head imaging compared with conventional gradient designs, will be worse than that of conventional designs for some body positions.

## 4 DISCUSSION AND CONCLUSIONS

The goal of this work was to develop new concepts and methods for minimum‐E‐field gradient design, and to establish an understanding of the limits to which E‐field can be reduced in gradient design, while maintaining desired imaging performance. The theoretical limits can then act as reference bounds to help assess the degree of optimality of practical designs and to guide design choices.

For transverse gradient coils designed for imaging the head, our design strategy and analysis shows that the controlled introduction of a concomitant field is the essential element in reducing E‐fields, with no appreciable changes to gradient distortion. The addition of the concomitant component reduces E‐field hotspots in the shoulder area, while increasing the E‐field on the head. A fully optimized design balances the maximum value of E‐field in these two body regions and can be achieved with modestly asymmetric transverse gradient designs.

Comparing the E_max_ results in Figure 2 for the real ESP gradient coil with the theoretical best (vertical asymptotes) for region BC, we predict that E_max_‐constrained solutions could yield E‐field reductions as much as about 34% for the X coil and about 22% for the Y coil. To reduce the E‐field, the curves in Figure 2 indicate that placing windings on the conical section in addition to the cylindrical primary section (acd) should be beneficial; this finding is consistent with previous literature.^8^, ^22^ There may be practical challenges; for example, allowing windings on the conical surface may lead to additional manufacturing complexity. Nonetheless, the methods described here can be used to help make rational tradeoff determinations for any real designs.

Perhaps the most dramatic result of our work is the realization that the minimum E‐field is relatively insensitive to the gradient coil inner diameter; this means that major gains in PNS performance can be achieved even for body‐sized gradient coils optimized for imaging the head. As shown by the A‐region blue curves of Figure 2, the vertical asymptote is virtually identical to the head‐only BC‐region asymptote, and Figure 3 shows remarkable E_max_ reductions of 1.8‐fold for the X coil and 2.8‐fold for the Y coil. This shows the potential to achieve head gradient levels of PNS performance without having to sacrifice extremely valuable patient bore space, although at the cost of increased magnetic energy and increased gradient amplifier power requirements compared with head gradients. The methods presented here will allow these optimal designs to be identified and rendered to practice with trade‐offs evaluated rationally and with reference to theoretical limits.

We note that a possible implementation option, similar to that proposed in previous literature,^20^ is the design of symmetric or asymmetric gradient windings with a separate winding intended to add or remove the concomitant field during head or body imaging; this independent control of main gradient and concomitant fields may permit more flexible control of E‐fields over a range of body positions. It would be straightforward to make these coils electrically orthogonal and torque‐balanced.

Applying the E_max_‐constrained gradient design concepts to body size coils, Figure 6 shows that even with a 40‐cm imaging region diameter, significant improvements in PNS performance (1.5‐fold to 3‐fold) are achievable for head imaging, although these major gains may reverse when imaging other body regions. Such large gains in PNS performance for head imaging may easily justify the trade‐offs for body imaging.

A limitation of our work is the use of cylindrical bounding regions A, B and C, which means that the theoretical minimum E‐field solutions described in this paper are not guaranteed to apply to geometries that do not fit within these build envelopes.^31^ In addition, we acknowledge that subject‐specific peripheral nerve stimulation will depend on characteristics of nerves (location, orientation, and other anatomical and physiological features) and 3D body composition that are not accounted for using our simplified methods. However, the methods described here, combined with our demonstrated ability to reasonably accurately predict population‐mean PNS thresholds using fast E‐field calculations and simplified body models that conform to existing safety standards,^17^ provide an attractive and practical approach to the long‐standing goal of PNS‐optimal gradient design.

The concept of constraining/minimizing E‐fields or controlling PNS performance by intelligent gradient design has been proposed in previous literature^18^, ^19^, ^20^, ^21^, ^22^, ^23^, ^24^, ^25^ and has inspired the present work. We believe our work contributes significant new insights and practical solutions to the problem of PNS‐optimal gradient design.

## Supporting information

Supplementary Material

## References

[1] Winkler SA , Schmitt F , Landes H , et al. Gradient and shim technologies for ultra high field MRI. Neuroimage. 2018;168:59‐70. 27915120 10.1016/j.neuroimage.2016.11.033 PMC5591082

[2] Zhang B , Yen YF , Chronik BA , McKinnon GC , Schaefer DJ , Rutt BK . Peripheral nerve stimulation properties of head and body gradient coils of various sizes. Magn Reson Med. 2003;50:50‐58. 12815678 10.1002/mrm.10508

[3] Bourland JD , Nyenhuis JA , Schaefer DJ . Physiologic effects of intense MR imaging gradient fields. Neuroimaging Clin N Am. 1999;9:363‐377. 10318720

[4] International Electrotechnical Commission . Medical electrical equipment—Part 2–33: Particular requirements for the basic safety and essential performance of magnetic resonance equipment for medical diagnosis. Edition 3.2. Geneva, Switzerland: IEC; 2015.

[5] Setsompop K , Kimmlingen R , Eberlein E , et al. Pushing the limits of in vivo diffusion MRI for the Human Connectome Project. Neuroimage. 2013;80:220‐233. 23707579 10.1016/j.neuroimage.2013.05.078 PMC3725309

[6] Harvey PR , Katznelson E . Modular gradient coil: a new concept in high‐performance whole‐body gradient coil design. Magn Reson Med. 1999;42:561‐570. 10467301 10.1002/(sici)1522-2594(199909)42:3<561::aid-mrm19>3.0.co;2-0

[7] Foo TKF , Laskaris E , Vermilyea M , et al. Lightweight, compact, and high‐performance 3T MR system for imaging the brain and extremities. Magn Reson Med. 2018;80:2232‐2245. 29536587 10.1002/mrm.27175 PMC6107412

[8] Tang F , Liu F , Freschi F , et al. An improved asymmetric gradient coil design for high‐resolution MRI head imaging. Phys Med Biol. 2016;61:8875‐8889. 27910827 10.1088/1361-6560/61/24/8875

[9] Vaughan T , DelaBarre L , Snyder C , et al. 9.4T human MRI: preliminary results. Magn Reson Med. 2006;56:1274‐1282. 17075852 10.1002/mrm.21073 PMC4406343

[10] Alsop DC , Connick TJ . Optimization of torque‐balanced asymmetric head gradient coils. Magn Reson Med. 1996;35:875‐886. 8744016 10.1002/mrm.1910350614

[11] Abduljalil AM , Aletras AH , Robitaille PM . Torque free asymmetric gradient coils for echo planar imaging. Magn Reson Med. 1994;31:450‐453. 8208122 10.1002/mrm.1910310415

[12] Roemer PB . Transverse gradient coils for imaging the head. USPTO, 1993, Patent No. 5177442A. Filed July 1, 1991.

[13] Bowtell RW , Mansfield P . Quiet transverse gradient coils: Lorentz force balanced designs using geometrical similitude. Magn Reson Med. 1995;34:494‐497. 7500892 10.1002/mrm.1910340331

[14] Lee S‐K , Mathieu J‐B , Graziani D , et al. Peripheral nerve stimulation characteristics of an asymmetric head‐only gradient coil compatible with a high‐channel‐count receiver array. Magn Reson Med. 2016;76:1939‐1950. 26628078 10.1002/mrm.26044 PMC4889567

[15] Wade T , Alejski A , McKenzie C , Rutt BK . Peripheral nerve stimulation thresholds of a high performance insertable head gradient coil. In: Proceedings of the 24th Annual Meeting of the ISMRM, Singapore, 2016. Abstract #3552.

[16] Davids M , Guerin B , Vom Endt A , Schad LR , Wald LL . Prediction of peripheral nerve stimulation thresholds of MRI gradient coils using coupled electromagnetic and neurodynamic simulations. Magn Reson Med. 2019;81:686‐701. 30094874 10.1002/mrm.27382 PMC6258337

[17] Roemer PB , Wade TP , Alejski A , McKenzie CA , Rutt BK . Electric field calculation and peripheral nerve stimulation prediction for head and body gradient coils. arXiv, 2020. arXiv:2012.08694. 10.1002/mrm.28853 PMC8651073 34080744

[18] Mansfield P , Haywood B . Controlled E‐field gradient coils for MRI. Phys Med Biol. 2008;53:1811‐1827. 18364540 10.1088/0031-9155/53/7/001

[19] Mansfield P , Bowley RM , Haywood B . Controlled E‐field gradient coils. MAGMA. 2003;16:113‐120. 14593514 10.1007/s10334-003-0015-7

[20] Hidalgo‐Tobon SS , Bencsik M , Bowtell R . Reducing peripheral nerve stimulation due to gradient switching using an additional uniform field coil. Magn Reson Med. 2011;66:1498‐1509. 21604293 10.1002/mrm.22926

[21] Sanchez‐Lopez H , Zilberti L , Bottauscio O , Chiampi M , Yang X , Xu Y . Controlled E‐peak field gradient coil. In: Proceedings of the 25th Annual Meeting of the ISMRM, Honolulu, Hawaii, 2017. Abstract #4333.

[22] Davids M , Guérin B , Schad LR , Wald LL . Peripheral nerve stimulation modeling for MRI. eMagRes. 2019;8:87‐101.

[23] Sanchez CC , Garcia SG , Power H . E‐coil: an inverse boundary element method for a quasi‐static problem. Phys Med Biol. 2010;55:3087‐3100. 20463375 10.1088/0031-9155/55/11/007

[24] Sanchez CC , Power H , Garcia SG , Bretones AR . Quasi‐static multi‐domain inverse boundary element method for MRI coil design with minimum induced E‐field. Eng Anal Boundary Elem. 2011;35:264‐272.

[25] Davids M , Guerin B , Klein V , Wald LL . Optimization of MRI gradient coils with explicit peripheral nerve stimulation constraints. IEEE Trans Med Imaging. 2021;40:129‐142. 32915730 10.1109/TMI.2020.3023329 PMC7772273

[26] Diffrient N , Tilley AR , Bardagjy JC . Humanscale 1/2/3 Manual. Chicago, IL: IA Collaborative Ventures LLC; 2017.

[27] Harrington RF . Time‐Harmonic Electromagnetic Fields. New York: McGraw‐Hill; 1961:480.

[28] Tan ET , Lee SK , Weavers PT , et al. High slew‐rate head‐only gradient for improving distortion in echo planar imaging: preliminary experience. J Magn Reson Imaging. 2016;44:653‐664. 26921117 10.1002/jmri.25210 PMC4983491

[29] Meier C , Zwanger M , Feiweier T , Porter D . Concomitant field terms for asymmetric gradient coils: consequences for diffusion, flow, and echo‐planar imaging. Magn Reson Med. 2008;60:128‐134. 18581353 10.1002/mrm.21615

[30] Tao S , Weavers PT , Trzasko JD , et al. Gradient pre‐emphasis to counteract first‐order concomitant fields on asymmetric MRI gradient systems. Magn Reson Med. 2017;77:2250‐2262. 27373901 10.1002/mrm.26315 PMC5217757

[31] Feldman RE , Hardy CJ , Aksel B , Schenck J , Chronik BA . Experimental determination of human peripheral nerve stimulation thresholds in a 3‐axis planar gradient system. Magn Reson Med. 2009;62:763‐770. 19526504 10.1002/mrm.22050

