# Supplementary material for: Minimum electric‐field gradient coil design: Theoretical limits and practical guidelines
Source: Magn Reson Med. 2021 Feb 9;86(1):569–80. doi: 10.1002/mrm.28681 (PMC8049068; doi:10.1002/mrm.28681)
Supplement: Supplementary file 1 — Supplementary Material [file MRM-86-569-s001.docx]

**Supporting Information**

**Proof that maximum** $\left| \boldsymbol{E} \right|$ **lies** **on surface of uniform-interior body model**

We wish to show here that the maximum of the electric field magnitude $\left| \boldsymbol{E} \right|$ lies on the surface of a region of uniform interior properties. This is equivalent to showing that there can be no maximum of $\left| \boldsymbol{E} \right|$, or equivalently of $\left| \boldsymbol{E} \right|^{2}$, anywhere within that region.

It is well known mathematically that a minimum or maximum of a multi-variable continuous function occurs at a critical point at which the resultant Jacobian (matrix of first order partial derivatives) has a rank that is less than the dimension of the matrix (at least one row of zeros). The Hessian matrix of partial second order derivatives then describes the curvature and is used to determine whether the critical point is a maximum, minimum or saddle point. A critical point is a maximum if the Hessian matrix is negative definite which in turn requires that all its eigenvalues be negative.

There are two other well-known mathematical properties to recognize about the Hessian: 1) the trace (sum of the diagonals) is the Laplacian; and, 2) the trace is numerically equal to the sum of the eigenvalues of the matrix. Hence, in order to show that there is no maximum of $\left| \boldsymbol{E} \right|^{2}$ in the region of interest, it is sufficient to show that $\nabla^{2}\left| \boldsymbol{E} \right|^{\boldsymbol{2}}$ is ≥ 0 everywhere over that region.

Expanding the Laplacian ${\nabla^{2}\left| \boldsymbol{E} \right|}^{2}$ we obtain

$\nabla^{2}\left| \boldsymbol{E} \right|^{\boldsymbol{2}}=2\left[ \left| \nabla E_{x} \right|^{2}+\left| \nabla E_{y} \right|^{2}+\left| \nabla E_{z} \right|^{2}+E_{x}\nabla^{2}E_{x}+E_{y}\nabla^{2}E_{y}+E_{z}\nabla^{2}E_{z} \right]$ (1)

We are operating in the quasi-static limit where time progresses slowly, and no charges accumulate inside a region of uniform properties. As a result, the divergence and curl of **E** are both zero and therefore the Laplacian of each component of **E** is zero, i.e.

$\nabla^{2}E_{x}=\nabla^{2}E_{y}=\nabla^{2}E_{z}=0$ (2)

Substitution of equation (2) into (1) we obtain

$\nabla^{2}\left| \boldsymbol{E} \right|^{\boldsymbol{2}}=2\left[ \left| \nabla E_{x} \right|^{2}+\left| \nabla E_{y} \right|^{2}+\left| \nabla E_{z} \right|^{2} \right]$ (3)

Since the right hand side of equation (3) contains a sum of quantities that are squared and must always be positive or zero, the trace of the Hessian matrix cannot be negative definite anywhere in the region and therefore there are no maxima of $\left| \boldsymbol{E} \right|^{\boldsymbol{2}}$ in a region of uniform properties.

Our simplified body models have uniform interior properties and the above proof guarantees that the maximum |E| will lie on the surface of such models.

We note that for non-uniform interior models constructed with multiple sub-regions, each with uniform properties, charges will build at the interfaces between regions. This means that the divergence of **E** will no longer be zero everywhere in the interior of the model. The global maximum |**E**| may then occur somewhere other than at the outer surface of the body.

**Choice of gradient coil basis functions**

The surface currents for the gradient coil are represented with a stream function ***S*** [1]. For a general surface in a curvilinear coordinate system, the stream function defines divergence-free current flow confined to the surface [2]

$$\boldsymbol{S}=s\left( u,v \right) \hat{\boldsymbol{n}}$$

where $s\left( u,v \right)$ is a scalar function in the surface coordinate (u, v) and $\hat{\boldsymbol{n}}$ is a unit vector normal to the surface. The surface current is given by the curl of $\boldsymbol{S}$**.**

$$\boldsymbol{J}=\nabla\times\boldsymbol{S}$$

The scalar $s\left( u,v \right)$ is constructed as a sum of basis functions in the 2D (u,v) coordinate system. For the gradient coil currents described here we use a circularly symmetric coordinate system with u as the coordinate in angle and v a coordinate that varies along the surface at constant angle of a cylindrical coordinate system. For a cylindrical surface, v is the simple z coordinate. On the other hand, for the surface described by region A of Figure 1, the v coordinate is chosen as the integrated distance around the circumference of region A at constant angle u.

The stream function is then chosen to be a 2D Fourier sum in the u and v coordinate where only 1 term is required in the u direction depending on gradient direction.

$$s\left( u,v \right)=\left\{ \begin{matrix} \cos\left( u \right) \\ \sin\left( u \right) \\ 1 \end{matrix} \right\}\sum_{m} \alpha_{m}\sin\left( m\frac{2\pi}{T} v \right)+\beta_{m}\cos\left( m\frac{2\pi}{T} v \right)$$

where $\alpha_{m}$ and $\beta_{m}$are the basis function unknowns and T is the distance around the circumference at constant angle. Typically, 50 to 100 terms in m are needed for full convergence.

**References**

1. Roemer, P.B. and Hickey, J.S., *Self-shielded gradient coils for nuclear magnetic resonance imaging.* USPTO, **1986** Patent no. 4737716 filed February 6 1986.

2. Hildebrand, F.B., *Advanced Calculus for Applications*. 2nd ed. **1976**, Englewood Cliffs, NJ: Prentice-Hall.
